# Antimicrobial, Mechanical and Thermal Studies of Silver Particle-Loaded Polyurethane

**DOI:** 10.3390/jfb4040358

**Published:** 2013-12-09

**Authors:** Deepen Paul, Sharmistha Paul, Nima Roohpour, Mark Wilks, Pankaj Vadgama

**Affiliations:** 1 School of Engineering and Materials Science, Queen Mary, University of London, London E1 4NS, UK; E-Mails: sharmisthapaul2003@yahoo.com (S.P.); n.roohpour@qmul.ac.uk (N.R.); 2 Barts Health Trust, Microbiology Department, London E1 2ES, UK; E-Mail: m.wilks@qmul.ac.uk

**Keywords:** polyurethane, silver, antibacterial, colony forming unit (CFU), stress-strain

## Abstract

Silver-particle-incorporated polyurethane films were evaluated for antimicrobial activity towards two different bacteria: *Escherichia coli* (*E. coli*) and *Staphylococcus aureus* (*S. aureus*). Distributed silver particles sourced from silver nitrate, silver lactate and preformed silver nanoparticles were mixed with polyurethane (PU) and variously characterized by field emission scanning electron microscopy (FESEM), fourier transform infra-red (FTIR) spectroscopy, X-ray diffraction (XRD) and contact angle measurement. Antibacterial activity against *E.coli* was confirmed for films loaded with 10% (w/w) AgNO_3_, 1% and 10% (w/w) Ag lactate and preformed Ag nanoparticles. All were active against *S. aureus*, but Ag nanoparticles loaded with PU had a minor effect. The apparent antibacterial performance of Ag lactate-loaded PU is better than other Ag ion-loaded films, revealed from the zone of inhibition study. The better performance of silver lactate-loaded PU was the likely result of a porous PU structure. FESEM and FTIR indicated direct interaction of silver with the PU backbone, and XRD patterns confirmed that face-centred cubic-type silver, representative of Ag metal, was present. Young’s modulus, tensile strength and the hardness of silver containing PU films were not adversely affected and possibly marginally increased with silver incorporation. Dynamic mechanical analysis (DMA) indicated greater thermal stability.

## 1. Introduction

Partially implanted medical devices become increasingly vulnerable with time to microbial colonization. A particularly common example of this is the intravascular infusion line, used for both acute and chronically ill patients. Maintained sterility here is vital for avoiding frequent line changes and for the safe administration of hydration fluids, electrolytes, drugs, nutrients and blood components. Regardless of the therapeutic benefits of intravascular devices, the escalating incidence of colonization and infection imposes a further, major therapeutic challenge [1]. The most common pathogens causing infection are Gram-positive *Staphylococcus aureus* (*S. aureus*) and *Staphylococcus epidermidis* and Gram-negative *Escherichia coli* (*E. coli*) and *Pseudomonas aeruginosa*, though infections tend to be more severe with *S. aureus* and *E. coli* [2]. Such nosocomial pathogens colonize both the outer and inner surfaces of catheters and are characteristically multi-antibiotic-resistant. They can also lead to bloodstream infection with high morbidity and mortality [3]. The prevention of catheter-related infection is becoming a high priority [4,5], particularly in view of the growing incidence of antibiotic-resistant organisms. Antibiotic loading of materials has been tried, but a substantial amount may be eluted in a first “burst”, posing a potentially serious toxic hazard [6].

*S. aureus* is a common cause of infection, its pathogenicity being partly due to coagulase production. This enables it to coagulate plasma in its microenvironment, helping to protect it from host defence mechanisms. A further, important feature of the bacterium has been its ability to develop resistance to commonly used antibiotics and also antiseptics [7,8,9]. Effectiveness cannot be guaranteed, even with a potent topical antibiotic, such as silver sulphadiazine [10], and alternatives are constantly needed. In this context, it is notable that mupirocin, whilst much more potent against *S. aureus*, does not provide universal coverage [11]. There is now an increased impetus to avoid indiscriminate antibiotics use to reduce the development of resistance organisms in clinical practice [12].

Polymer-silver combinations have been reported for a range of non-medical applications, where their electrical conductivity, light scattering and catalytic activity have proven to be valuable [13,14,15,16]. For medicine, the antimicrobial properties of silver (Ag) hold considerable promise; the mechanism of action, the development of bacterial resistance, toxicology and clinical utility have been reviewed extensively [17,18,19,20,21]. Ag has one of the highest levels of toxicity for microorganisms, but the least toxicity for eukaryotic cells [22]. The antimicrobial spectrum of Ag is exceptionally broad, and there is also significant virucidal activity [23]. A sufficient concentration of free silver ions is required, and whilst water soluble silver salts can give the necessary high concentrations, this is countered by sequestration by protein and other macromolecules. Loss through insoluble AgCl formation and chelation to microbial products is also a significant problem. Preformed Ag particles may be used, and here, the smaller the particle and the greater the relative surface area, the more efficient the antibacterial activity [24], most probably the result of enhanced silver ion release. Furno *et al.* [25] were able to link silver ion release from silver nanoparticles in a silicone to antimicrobial activity.

Resistance to the antimicrobial activity of Ag has been reported for some microorganisms [26]. Thus, in the case of the filamentous fungi, *Phoma sp*. 3.2883, *Phoma* PT35, *Fusariumoxysporum* andthe bacterium *Bacillus megatherium*, the adsorption and accumulation of Ag ion without an adverse effect are observed, and indeed, such organisms have been used in the reduction of environmental pollution and the recovery of Ag from environmental wastes [27,28,29].

Polyurethanes (PUs) comprise an important polymer group, used in industry as coatings, adhesives, foams, rubbers and composites. More recently, shape memory materials based on PU have been investigated and detailed structure and property correlations derived [30,31,32]. With their evident biocompatibility, they have also become useful implant materials. A key objective has been to improve their resistance to mechanical deformation without sacrificing elasticity and biocompatibility; their combination of tensile strength and Young’s modulus, whilst maintaining high elongation at break, is promising in this regard [33].

Here, we report on the structural, mechanical and thermal properties of PU, loaded with Ag salt and Ag nanoparticles, along with resultant antimicrobial activity.

## 2. Results and Discussion

Silver is less prone to microbial resistance than antibiotics, especially if rapid bactericidal action is achieved [34]. Relatively low concentrations of silver ion are needed, possibly because of active uptake and concentration by microorganisms. Organic components in biological fluids can significantly diminish the effectiveness [35], and concentrations as high as 0.56 mM have been proposed [36,37]. Cell membrane and solution proteins, for example, present nucleophiles and coordinating groups, such as sulfhydryls, hydroxyls and amines for silver. Once silver does bind to microbial cells, it denatures crucial proteins, disrupts DNA and RNA, inhibits cell replication and, ultimately, causes cell death. Silver can also displace other bound metal cations essential to cell survival. Accordingly, ionic silver is active against a range of pathogenic organisms, subject to complexation effects [38].

### 2.1. Characterization of PU-Ag Composites

#### 2.1.1. Structure

**Figure 1:**
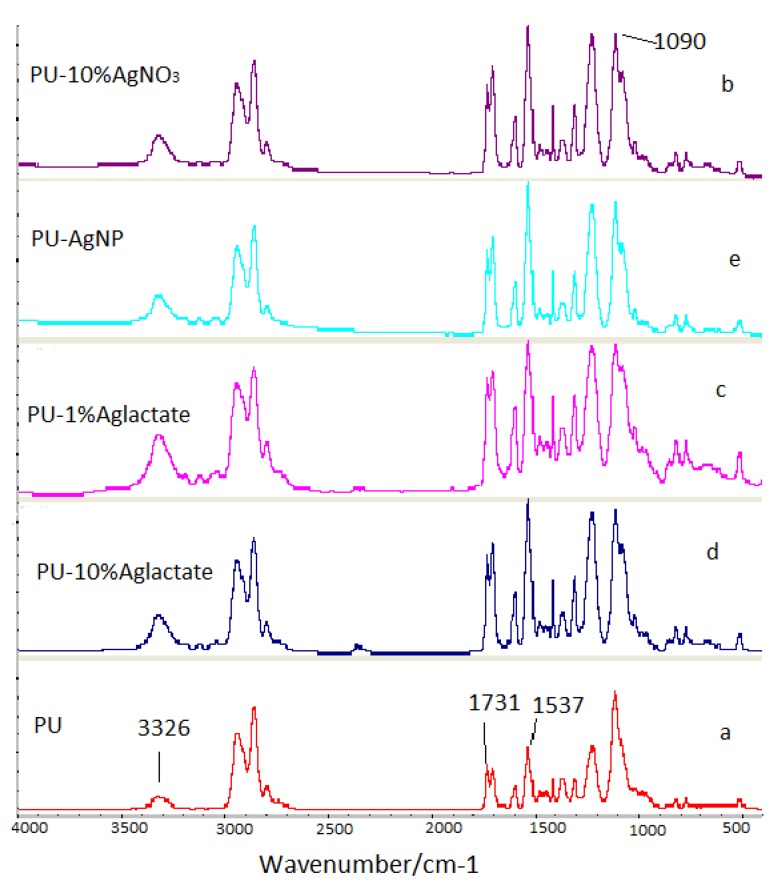
Fourier Transform Infra-red (FTIR) spectra of polyurethane (**a**) unmodified; and loaded with (**b**) 10% (w/w) AgNO_3_; (**c**) 1% (w/w) Ag lactate; (**d**) 10% (w/w) Ag lactate; and (**e**) Ag nanoparticles.

Figure 1 shows the fourier transform infra-red (FTIR) spectra of PU and PU-Ag composites between 400 and 4000 cm^−1^. These reveal broad, but consistent, structural effects of silver in PU. Absorption bands at 1703 and 1731 cm^−1^ are characteristic of a carbonyl group stretch; the former peak corresponds to carbonyl that is hydrogen bonded with the –NH groups of a neighbouring hard segment, whereas the latter is due to non-hydrogen bonded carbonyls within a soft segment. The matching peak ratio for pure PU is altered to varying degrees for the silver-loaded PU materials with an increase in the high frequency stretch. Peaks at 1310 and 1537 cm^−1^ are due to the NH– and C–N stretches, respectively, and are enhanced in the silver-loaded PU. The peak at 1090 cm^−1^ (C–O–C, aliphatic ether stretching) has a shoulder, and the broad peak at 3326 cm^−1^ (−NH stretching) is increased [39]. Overall, the spectral perturbations indicate that silver interacts with –N– and –O–, as might be expected from their electronegative properties.

**Figure 2:**
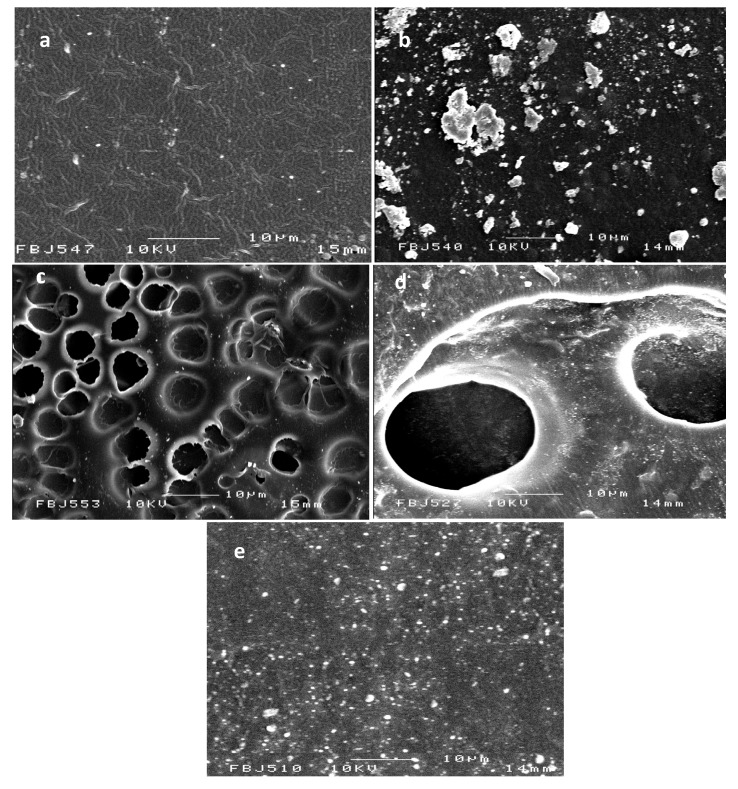
Field emission scanning electron microscopy (FESEM) images of polyurethane (**a**) unmodified; and loaded with (**b**) 10% (w/w) AgNO_3_; (**c**) 1% (w/w) Ag lactate;(**d**) 10% (w/w) Ag lactate; and (**e**) Ag nanoparticles.

The field emission scanning electron microscopy (FESEM) micrographs of PU and PU-Ag films are shown in Figure 2. PU under our casting conditions formed a dense fibrillary structure with fibril diameters of ~300 nm. The PU-Ag lactate film shows a porous structure with increased pore sizes at the higher Ag-lactate level; the surfaces are also covered with ~400nm Ag particles (Figure 2d). The porosity here may have been due to phase separation during preparation. AgNO_3_ tended to aggregated in the polymer, and aggregated silver particles with a highly variable cluster size (1–10 μm) are seen on the PU-AgNO_3_ (Figure 2b). A high surface coverage is seen for the PU-Ag nanoparticles (Figure 2e); solvent exposure was likely to have led to sub-surface particle penetration. Ag nanoparticles (AgNPs) of increased size ranging from 30 to 290 nm were coated on the PU-AgNPs composite film compared to the original 30–40 nm [40].

**Figure 3:**
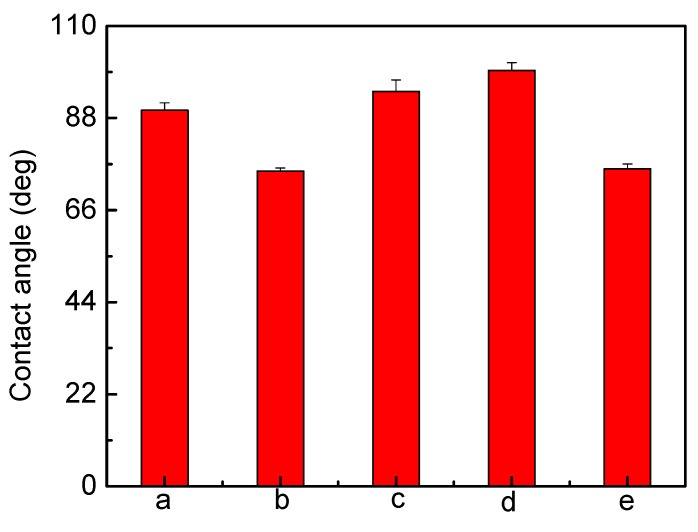
Contact angle measurements for polyurethane (**a**) unmodified polyurethane; and incorporated (**b**) 10% (w/w) AgNO_3_; (**c**) 1% (w/w) Ag lactate; (**d**) 10% (w/w) Ag lactate; and (**e**) Ag nanoparticles.

Except for Ag-lactate, the contact angle for PU decreased from 89.8° to 75.9° after Ag loading, indicating a more hydrophilic surface (Figure 3). Values of the contact angles were determined from fitting of the captured drop profile to the Young–Laplace Equation. The reproducibility of contact angles measurements was within ±5° or better. The PU used here was initially hydrophobic, and the greater hydrophilicity confirmed the presence of surface silver. In principle, more hydrophilic surfaces are advantageous with regard to blood compatibility [41,42]. The apparent increased hydrophobicity with Ag lactate-loaded material was the result of increased surface roughness (Figure 2).

**Figure 4:**
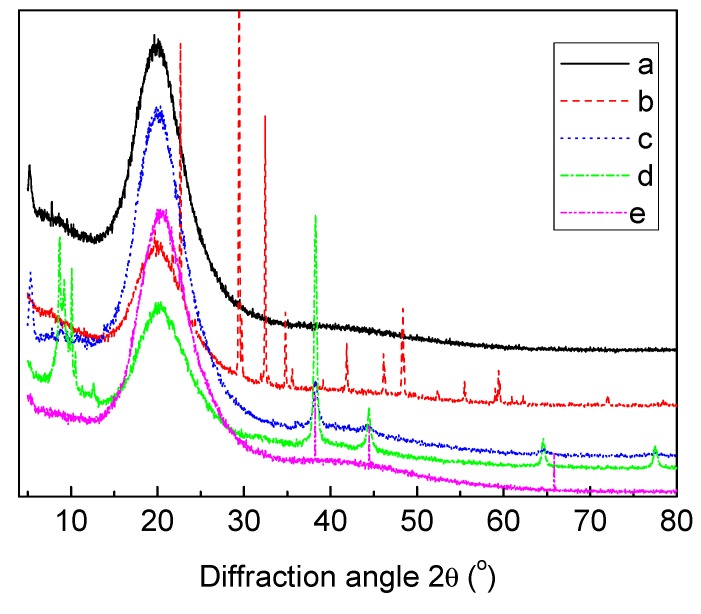
X-ray Diffraction curves for polyurethane (**a**) unmodified; and loaded with (**b**) 10% (w/w) AgNO_3_; (**c**) 1% (w/w) Ag lactate; (**d**) 10% (w/w) Ag lactate; and (**e**) Ag nanoparticles.

The X-ray Diffraction (XRD) patterns of pure PU, PU-10% (w/w) AgNO_3_, PU-1% (w/w) Ag lactate, PU-10% (w/w) Ag lactate and PU-Ag nanoparticles are shown in Figure 4. The diffraction peak near 2θ =20.26° is due to the hard segments in the PU, with Ag nanoparticles generating the sharp peaks seen at 2θ values of 38.20°, 44.45° and 65.89°, indicative of crystalline silver. Peaks seen in PU-Ag lactate at 2θ values of 8.72°, 38.23°, 44.45°, 64.57° and 77.37° and in PU-AgNO_3_ at 2θ values of 22.66°, 29.44°, 34.80°, 41.88°, 46.13°, 48.40°, 55.48° and 59.39° are also consistent with the presence of Ag metal in composites made using silver salt. The peak shifts in the PU-Ag nanoparticle composite may have been due to the formation of different lattice planes of face-centred cubic silver. 

A different distribution of silver in the polyurethane-silver composites may account for slight differences in the observed peaks for each formulation, but the presence of hard segments appears unaffected. A decrease in the number of hydrogen bonds between the soft and hard segments of polyurethane might be expected, decreasing crystallinity, consistent with such an observation on polypropylene containing micro- and nano-scale silver powders [43]. The peaks at, or near, 2θ values of 38.2°, 44.45° and 64.57° for all the composites are from the {111}, {200} and {220} lattice planes, of face-centred cubic silver, and indicate that the particles formed from the salts are silver metal, consistent with the top crystal plane reported previously [44,45,46]. Overall, the XRD and FTIR results indicate that the polymer chains interacted with embedded silver. 

**Figure 5:**
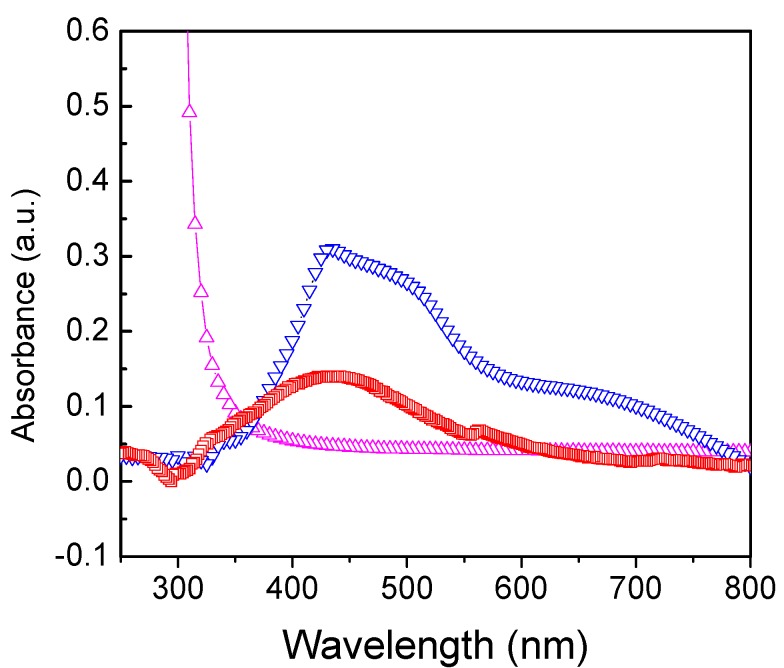
UV-Vis absorption spectra of PU-AgNP film (▽) along with PU film (Δ) and 12.46μg/ml AgNP solution (□).

The UV-Vis spectra of pure PU, PU-AgNP film along with preformed AgNP solution are shown in Figure 5. The colour of the PU films changed from colourless to yellow when coated with Ag NPs. The resultant broad spectrum of PU-AgNP film compared to AgNP solution may be due to the aggregation of AgNPs, showing a surface Plasmon resonance (SPR) peak at λ_max_ = 435 nm along with a shoulder at 500 nm and a new peak at 665 nm.

#### 2.1.2. Analysis of Mechanical Properties

**Figure 6:**
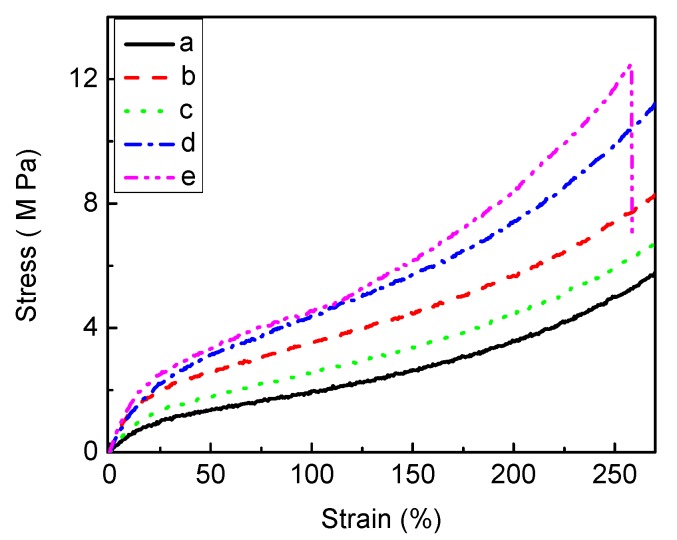
The stress-strain curves recorded for polyurethane (**a**) unmodified; and loaded with (**b**) 10% (w/w) AgNO_3_; (**c**) 1% (w/w) Ag lactate; (**d**) 10% (w/w) Ag lactate; and (**e**) Ag nanoparticles.

**Figure 7:**
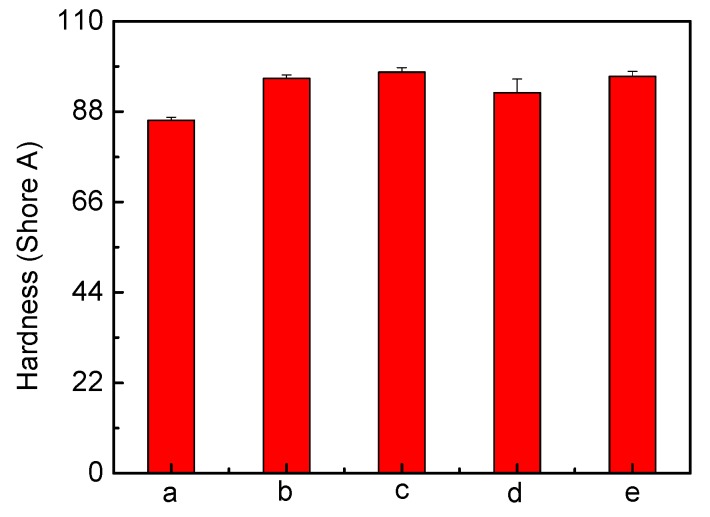
Hardness measurements of polyurethane (**a**) unmodified; and loaded with (**b**) 10% (w/w) AgNO_3_; (**c**) 1% (w/w) Ag lactate; (**d**) 10% (w/w) Ag lactate; and (**e**) Ag nanoparticles.

**Figure 8:**
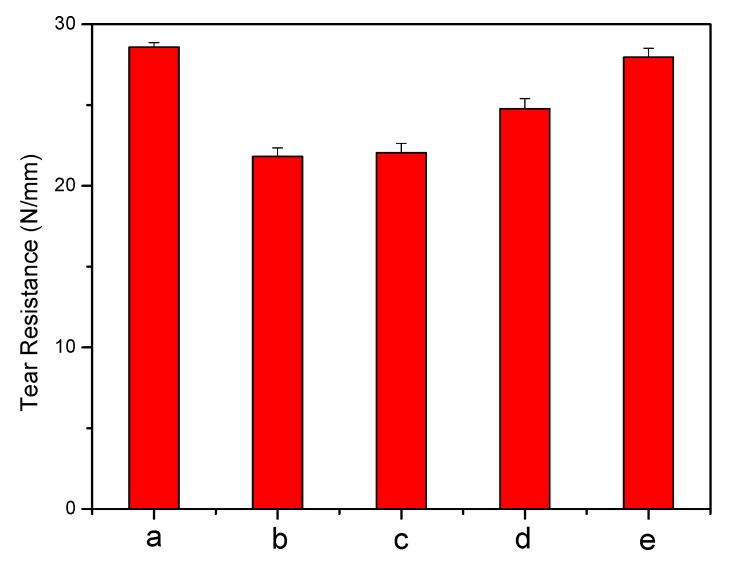
Tear resistance measurements of polyurethane (**a**) unmodified; and loaded with (**b**) 10% (w/w) AgNO_3_; (**c**) 1% (w/w) Ag lactate; (**d**) 10% (w/w) Ag lactate; (**e**) Ag nanoparticles.

To assess the effect of silver on the PU mechanical properties, tensile testing was undertaken (Figure 6). A change in the stress-strain behaviour after the introduction of silver can be seen. Stress increases in the modified PU films, which retain a clearly distinguishable yield point. Ag has led to slightly increased Young’s modulus and tensile strength. The hardness of modified PU also increased from 85.92 shore A (unmodified PU) up to 97.62 shore (Figure 7), indicating matrix reinforcement [14,47]. Tear resistance, expressed as the maximum force needed to tear a film (Figure 8) shows that a force of 28.59 N/mm is required for pure PU; the value decreases for all silver-incorporated materials, maximally for PU-10% (w/w) AgNO_3_, but is almost unchanged for PU-Ag nanoparticles, consistent with the report by Chou *et.al.* [47] and the superficial deposition of these particles in the PU. One effect of tear resistance may have been the generation of inter-locks or stress concentrating points by silver, leading to earlier failure [41]. The maintenance of mechanical properties suggests that the silver incorporation is feasible, without eroding the optimized properties of a base polymer.

#### 2.1.3. Analysis of Thermal Property

**Figure 9:**
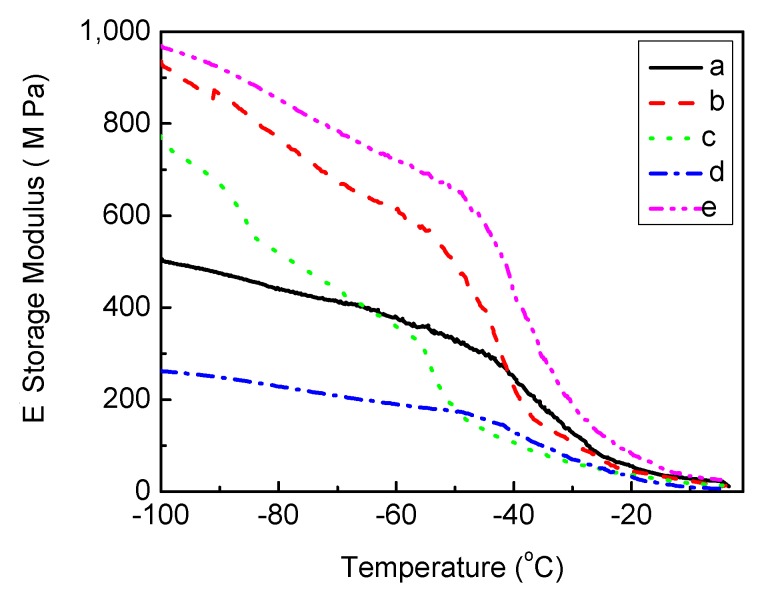
Temperature dependence of the storage modulus of polyurethane (**a**) unmodified; and loaded with (**b**) 10% (w/w) AgNO_3_; (**c**) 1% (w/w) Ag lactate; (**d**) 10% (w/w) Ag lactate; and (**e**) Ag nanoparticles.

**Figure 10:**
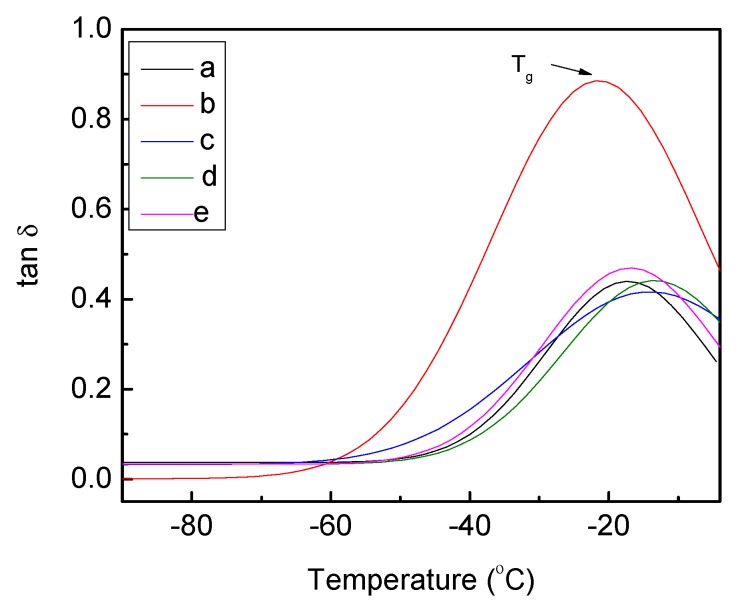
Temperature dependence of the tanδ of polyurethane (**a**) unmodified; and loaded with (**b**) 10% (w/w) AgNO_3_; (**c**) 1% (w/w) Ag lactate; (**d**) 10% (w/w) Ag lactate; and (**e**) Ag nanoparticles.

The storage modulus E' (energy stored during deformation due to stress) and damping factor tanδ (the ratio of energy dissipated and energy stored during deformation) of PU and PU-silver composites as a function of temperature are shown in Figure 9 and Figure 10, respectively. It can be seen that E' increases for different Ag composites, except 10% Ag-lactate, due presumably to its porous nature (Figure 2). The tanδ peak is associated with the soft segment glass transition temperature (T_g_). The introduction of Ag particles resulted in a slight increase in T_g_ and damping capacity in all composite films, except 10% AgNO_3_-PU.Well-dispersed Ag will restrict molecular motion, and this could have led to an increase in T_g_. In the case of 10% AgNO_3_-PU, micro-scale phase separation could have reduced hard segment content in the soft phase [48], decreasing T_g_ from pure PU (−5 °C from Figure 10). A T_g_ shift to a higher temperature has also been seen in polyvinyl alcohol with the addition of Ag particles [14].

### 2.2. Antimicrobial Activity Test of PU-Ag Composites

**Figure 11:**
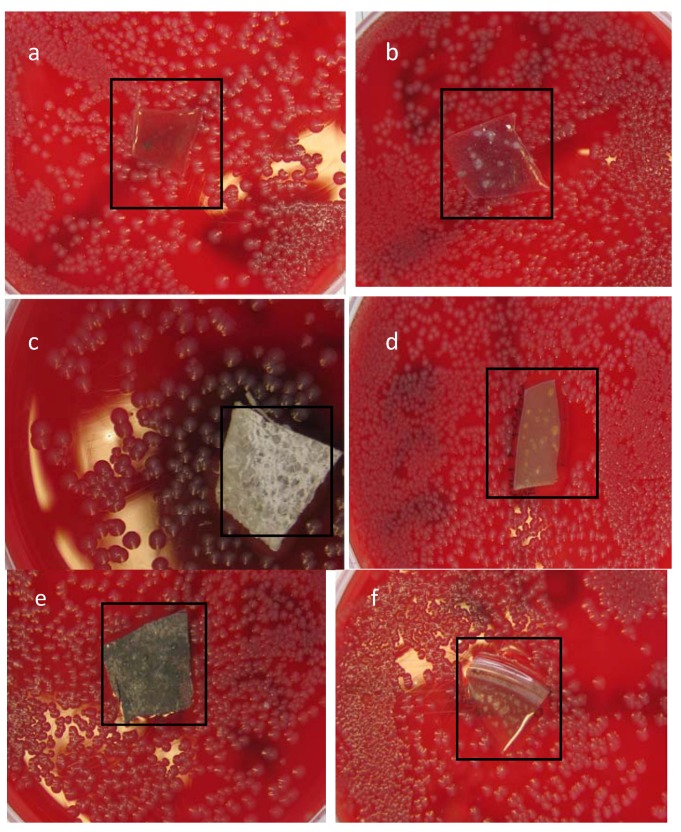
*E. coli* grown on iso-sensitest agar supplemented with 5% (v/v) defibrinated horse blood and incubated with (**a**) unmodified polyurethane; and polyurethane loaded with (**b**) 1% (w/w) AgNO_3_; (**c**) 10% (w/w) AgNO_3_; (**d**) 1% (w/w) Ag lactate; (**e**) 10% (w/w) Ag lactate; and (**f**) Ag nanoparticles.

When antibacterial activity was assessed for *E. coli* (Figure 11),there was a growth inhibition zone around PU loaded with 10% (w/w) AgNO_3_, 1%, 10% (w/w) Ag lactate and Ag nanoparticles. No inhibition was seen around unmodified polyurethane film or polyurethane loaded with 1% (w/w) AgNO_3._ The lack of activity of the latter was presumably due to lower Ag^+^ release, and this film was not used for further study. The effectiveness of 1% (w/w) silver lactate was likely to have been due to the higher surface area of the associated porous PU (Figure 2). 

**Figure 12:**
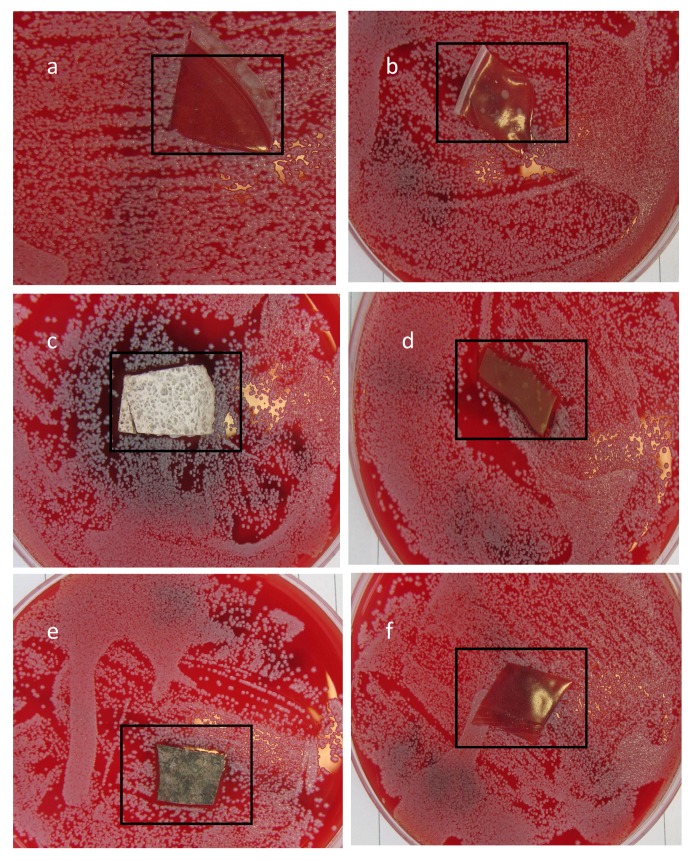
*S. aureus* grown on iso-sensitest agar supplemented with 5% (v/v) defibrinated horse blood and incubated with (**a**) unmodified polyurethane; and polyurethane loaded with (**b**) 1% (w/w) AgNO_3_; (**c**) 10% (w/w) AgNO_3_; (**d**) 1% (w/w) Ag lactate; (**e**) 10% (w/w) Ag lactate; and (**f**) Ag nanoparticles.

Film activity was also seen against *S. aureus*, but the film coated with preformed Ag nanoparticles (Figure 12) showed only a minor effect. With the latter, particle loading and size will have been relevant to silver ion release; this has implications for materials designed for clinical use. The zone of inhibition (ZOI) was determined on each side of a polymer film for *E. coli* and *S. aureus* and measured mean values are reported in Table 1 and Table 2 respectively. The greater resistance of *S. aureus* may have been due to its thick peptidoglycan walls, reducing the penetration of Ag^+^, while the lipopolysaccharide walls of Gram-negative bacteria likely provided an electrostatically attractive surface [49].

**Table 1:** Zone of inhibition (ZOI) for different PU-Ag composite films for *E. coli.*

Film type	*E. coli*	
	Average ZOI (mm)	Haemolysis effect
1% AgNO_3_	1.5	none
10% AgNO_3_	3.0	wide effect
1%Aglactate	5.0	yes
10% Aglactate	4.0	yes
Ag nanoparticles	3.0	yes

**Table 2:** Zone of inhibition (ZOI) for different PU-Ag composite films for *S. aureus*.

Film type	*S. aureus*	
	Average ZOI (mm)	Haemolysis effect
1% AgNO_3_	none	none
10% AgNO_3_	1.5	wide effect
1% Aglactate	2.5	yes
10% Aglactate	2.0	yes
Ag nanoparticles	minor	none

## 3. Experimental Section

### 3.1. Materials

Two bacterial strains, namely *E. coli* (NCTC 10148) and *S. aureus* (NCTC 6571), were used in the microbial study. Luria-Bertani (LB) medium (Oxoid, Basingstoke, UK) was used to grow and maintain the bacterial cultures, and the zone of inhibition was studied with iso-sensitest agar (ISA; Oxoid, Basingstoke, UK) supplemented with 5% (v/v) defibrinated horse blood. Silver nitrate, silver lactate, sodium citrate tribasic dehydrate, 4,4'-methylene bis(phenylisocyanate) (MDI), polytetramethylene glycol (PTMO, Mw 1000 g/mol), 1,4-butandiol (BD), tetrahydrofuran (THF), *N*,*N*-dimethylformamide (DMF) were of the highest purity available (Sigma) and used as received without further purification.

### 3.2. Synthesis of Polyurethane

Polyurethane was synthesized from its monomer, MDI and PTMO by a two-step process, using BD as a chain extender. First, the pre-polymer was prepared from a reaction of 5.005 g of MDI in 40 mL of DMF and 10 g of PTMO in 20 mL of DMF in a 500-mL four-neck cylindrical vessel heated to 60 °C for 90 min with mechanical stirring under a nitrogen atmosphere. Subsequently, 0.92 g of BD in 10 mL DMF was added slowly to the prepolymer at 110 °C over 240 min and the mixture allowed to react fully. The molar ratio used was 1:2:1 for MDI:PTMO:BD, which was consistent with providing a 67% soft segment polymer. The polyurethane product was finally washed with Milli-Q water (18 MΩ∙cm) then methanol and in an oven at 80 °C [50,51].

### 3.3. Preparation of Silver Nanoparticles

Five-hundred millilitres of 1 mM silver nitrate solution in distilled water was heated to boiling. Then, 20 mL of 1% (w/v) sodium citrate solution was added, and boiling continued until a pale yellow solution was obtained [52]. The solution was cooled to room temperature, and Ag nanoparticles were harvested using a previously reported procedure [52] after the addition of THF as a stabilizer [40].

### 3.4. Preparation of Polyurethane and Polyurethane-Ag Composites Films

One gram of PU was dissolved in 25 mL THF, and after stirring until homogeneous, 25 mL of the polymer solution was cast in a glass Petri dish of a 96-mm diameter. The polyurethane-only films were allowed to dry at room temperature for two days; then, they were removed for further characterization. 

Composite films of different silver content were readily obtained using a solvent casting method [14]. For the of PU-AgNO_3_ films, 1 g of polyurethane was dissolved in 25 mL of THF, and after stirring until homogeneous, 25 mL of the solution was mixed with 0.3 and 3 mL, respectively, of 3.33% (w/v) silver nitrate in aqueous solution to produce polyurethane containing 1% and 10% (w/w) AgNO_3_. For the PU-Ag lactate film, 0.2 and 2 mL of 5% silver lactate aqueous solution were mixed with 25 mL of polymer solution to produce polyurethane containing 1% and 10% (w/w) Ag-lactate. Mixtures were cast in Petri dishes of a 96-mm diameter, and the films were allowed to dry at room temperature for two days.

A polyurethane film of a 96-mm diameter was soaked in 520 mL of an aqueous suspension of silver nanoparticles (100 μg/mL). One millilitre of THF was added, and the mixture was kept in closed glass vials overnight to promote the coverage of the surface. THF served as a stabilizer and prevented the formation of larger nanoparticles [40]. The film was washed several times with Milli-Q water to remove any absorbed citrate and air-dried. 

### 3.5. Preparation of Inoculum

*E. coli* and *S. aureus* were selected as target indicators for antimicrobial activity. Strains for the control were stored at −70 °C on beads in glycerol broth. From the beads, the strains were subcultured in LB medium every week. From this pure culture, touching at least four morphologically similar colonies, the culture was transferred into iso-sensitest broth supplemented with 5% (v/v) defibrinated horse blood. The bacteria were grown aerobically at 37 °C for 18 h. Visible turbidity equal to 0.5 McFarland Standards (BioMérieux, Basingstoke, UK) was achieved by adding sterile distilled water. To aid visual comparison, a white background with a contrasting black line was used for inspection. The culture was finalized by 1:100 and 1:10 dilution in sterile distilled water before inoculation of either *E. coli* or *S. aureus* [53].

### 3.6. Zone of Inhibition

For the zone inhibition study, 25 mL of sterile iso-sensitest agar, according to the manufacturer’s instructions, was poured into 90-mm disposable, sterilized Petri dishes supplemented with 5% (v/v) defibrinated horse blood and allowed to solidify. The plates were stored at 4–8 °C in sealed plastic bags. Ten microlitres of bacterial water were streaked over a plate and spread uniformly using a 6-cm sterile needle. PU pieces were then gently placed over the solidified agar in different Petri dishes. Incubation times were 24 h at 37 °C.

### 3.7. Film Characterization

For structure analysis, the FTIR spectra of samples were recorded on an FTIR spectrometer (Nicolet 8700 FTIR, Thermo Electron Corporation, Hertfortshire, UK) and spectra collected from 400 to 4000 cm^−1^, with 4 cm^−1^ resolution over 128 scans. For the FESEM study, a Jeol JSM 6300F instrument (Tokyo, Japan) recorded gray scale images with 8-bit resolution at different magnifications at a primary electron beam energy of 10 kV operated at a working distance of 15 mm. Ultrasonically-cleaned unmodified and modified PU with different Ag loadings were made conductive with an ultrathin layer of gold deposited by sputtering before the FESEM measurements.

Contact angle measurement was undertaken using a CAM 200 model, KSV instrument using droplets of 2 μL of double distilled water dispensed on film on a glass substrate. Five sets of measurements were made to derive the mean and standard deviation. The X-ray Diffraction patterns of samples were recorded with an Xpert-Pro X-ray diffractometer (PANalytical, Almelo, the Netherlands) employing a scanning rate of 0.03° /min from 5° to 120° with Cu Kα irradiation (45KV, 30 mA; the wavelengths of Cu Kα_1_ and Cu Kα_2_ are 1.540598Å and 1.54442Å, ratio 2:1).

For mechanical testing, tensile strength was determined using an Instron Universal Testing machine (model No 5584, Instron Co., Macclesfield, UK) at room temperature. Rectangular specimens (60 mm × 6 mm) were stretched until breaking at a crosshead rate of 20 mm∙min^−1^; stress-strain curves were recorded. Hardness and tear resistance were respectively determined according to ASTM D2240-05 [54] and ASTM D1004-66 standards [55] using an H17 Shore Scale Hardness Tester (H.W. Wallace & Co. Ltd., Croydon, UK) and an Instron Universal Testing machine (model No. 5584). The data represents the mean values of five independent measurements.

To test thermal properties, Dynamic Mechanical Analysis (DMA) was performed on a Dynamic Mechanical Thermal Analyser DMA Q 800 at a frequency of 1 Hz, an amplitude of 15 μm and a static force of 0.01 Newtons. Samples were kept under isothermal conditions at −100 °C for 2 min and heated from −100 to 0 °C at a heating rate of 5 °C∙min^−1^. The sample used had a dimension of 20 mm in length, 5.3 mm in width and 0.14 mm in thickness. The data presented represent the mean of three independent measurements.

## 4. Conclusions

Polyurethane, despite its hydrophobic nature, is hygroscopic and so can take up silver salt solution for composite formation. The later interaction with an external solution can then release bactericidal concentrations of silver ion with an evident effect on *E. coli* and *S. aureus*. In this study, the method used to detect antibacterial activity was relatively insensitive, relying on gross inhibition of an inoculum visible to the naked eye. *In vivo*, the scale of initial bacterial loading would be expected to be much lower, and inhibition effects might be more pronounced than observed here. The components used here are cheap; chemical derivatization is not required, and loading can be varied, making the method simple to apply. The avoidance of mechanical compromise is also an advantage. There are clearly multiple routes to silver addition, and further work is warranted on their different release dynamics and, also, on the resistance to surface film formation, a distinct modality of the remote inhibition of growth through diffusive release of silver ions. The silver composite films have improved marginally mechanical properties and greater thermal stability, but the feasibility of silver loading for such an application would need further evaluation. 
